# Editorial: Community series in autoimmune diabetes: molecular mechanisms and neoantigens, volume II

**DOI:** 10.3389/fimmu.2025.1631976

**Published:** 2025-06-06

**Authors:** Arnaud Zaldumbide, Sally C. Kent

**Affiliations:** ^1^ Department of Cell and Chemical Biology, Leiden University Medical Center, Leiden, Netherlands; ^2^ Diabetes Center of Excellence, Department of Medicine, University of Massachusetts Chan Medical School, Worcester, MA, United States

**Keywords:** type 1 diabetes, beta cell stressors, inflammation, neoepitopes, T cell responses

With the advent of teplizumab, which delays the onset of type 1 diabetes (T1D) for those who have stage 2 diabetes (two detectable autoantibodies with abnormally high levels of blood glucose), we now have one treatment to benefit those individuals, but we still have an incomplete understanding of the disease process. We now favor the idea that physiological/metabolic stress on the insulin-producing beta cell is a major contributor to beta cell dysfunction and triggering of the autoreactivity that leads to beta cell death, but we do not understand all factors in what appears to be a multifaceted process of T1D development. We have now identified several stress processes that alter the beta cell function as well as induce the formation of neo-epitopes that may significantly contribute to the autoreactive attack of T cells on the stressed beta cells. While the development of therapies targeting beta-cell dysfunction/destruction and the autoimmune attack for T1D remains challenging, this increased understanding of these processes in individuals at risk for or with T1D is beneficial.

In this Research Topic, in one form of beta-cell stress, Muñoz García et al. highlighted the importance of the islet microenvironment in the amplification of the inflammation during T1D progression, showing that a strong chemokine signature (CXCL1, CXCL2, CXCL3, and CXCL10) is found from the ductal cells and cells expressing a ductal-acinar cell phenotype from control islets treated with inflammatory cytokines and from islets from two donors with T1D. Interestingly, they found that CXCL8 expressed by endocrine cells may have a functional effect on beta cells by decreasing insulin secretion. This propagation of the inflammatory signals has also been illustrated by Dekkers et al., who showed that knockdown of Heat Shock Protein Family A (*HSPA5)*, which encodes BiP/GRP78 in a beta-cell line, resulted in upregulation of pathways in the unfolded protein response (UPR), an ER stress response, as well as changing the cargo of extracellular vesicles (EVs) and decreasing miRNAs involved in IL-1β signaling coming from the beta cell line. These EVs activated monocytes, indicating a role in the autoimmune response of stressed beta cells.

In another stress model, Austin et al. illustrated the importance of adaptive mechanisms to stress and autophagy in particular, showing a connection between a critical autophagy enzyme, ATG7, and the incidence of diabetes development in B6 mice. Indeed, in these mice, the ATG7 beta cell-specific knock out resulted in upregulation of genes involved in inflammation, ER stress, and the ER-associated degradation pathway and in the upregulation of MHC-I on beta cells and on CD45+ cells with increased T -cell activation. These data indicate that impaired beta cell autophagy leads to beta cell stress and alterations of the beta cell associated with autoimmune responses. Similarly, in a cautionary report, van Tienhoven et al. established a link between the molecular mechanism involved in cancer and T1D development, demonstrating that checkpoint inhibitor treatment of cancer patients who then present with T1D show a priming of the T cell autoimmune response to Ins-DRiP (a neoepitope, insulin defective ribosomal product induced by ER stress) and other islet-associated epitopes, whereas no reactivity was detected from the periphery of these patients prior to checkpoint inhibitor treatment.

In addition to Ins-DRiP as a neoepitope, there are many other categories of neoepitopes that can be induced by other stressors. Post-translationally modified epitopes (PTMs) are a rich area for T cell autoreactive targets in T1D. In Alhamar et al., the authors review the role of oxidative post-translational modification of epitopes along with other possible PTMs. They highlight that accumulation of reactive oxygen species (ROS) and an insufficient antioxidant response in the beta cell creates a perfect environment for oxidative stress and oxidation of proteins. The authors feature the autoreactive T cell and autoantibody oxidated PTMs found for insulin and glutatmic acid decarboxylase 65 (GAD65) in T1D as well as for collagen type II in rheumatoid arthritis (RA). The authors also highlight the opportunities in therapies to modulate the immune response to these PTMs. In Wenzlau et al., the authors examine another category of PTM neoepitope, the hybrid insulin peptide (HIP), which is formed by the fusion of a fragment of insulin with another fragment of a beta cell granule protein. Here, the authors use a monoclonal antibody (mAb) generated to the HIP of an insulin C-peptide fragment fused to a cleavage product of pro-islet amyloid polypeptide (6.9HIP) which the murine BDC-6.9 T cell clone recognizes. They used this mAb to follow HIP formation in the islets of the NOD mouse during disease progression and found that the mAb detected the 6.9HIP only in insulin-positive beta cells and in intra-islet antigen-presenting cells in infiltrated islets in advanced disease. Exposing NOD islets to ER stress with tunicamycin increased levels of 6.9HIP in crinosomes and dense-core granules from the beta cells.

Finally, as a reminder that therapies may not be effective for all manifestations of the autoimmune disease and to understand the functions of all immune cell populations in autoimmune diseases, Marasco et al. show that reductions in memory and switched memory B cells and significant expansions of CXCR5^-^ PD1^+^ T peripheral helper cells (Tph cells) correlate with RA patients with bone erosions on disease-modifying anti-rheumatic drugs (DMARDS).

Altogether, the different studies presented in this Research Topic provide novel insights into the role of cellular stress and its consequences in the development of autoimmunity.

